# Rapamycin/sodium hyaluronate binding on nano-hydroxyapatite coated titanium surface improves MC3T3-E1 osteogenesis

**DOI:** 10.1371/journal.pone.0171693

**Published:** 2017-02-09

**Authors:** Chao Liu, Jian Yong Dong, Lin Lin Yue, Shao Hua Liu, Yi Wan, Hong Liu, Wan Ye Tan, Qian Qian Guo, Dong Zhang

**Affiliations:** 1 Department of Oral and Maxillofacial Surgery and Institute of Dental Medicine, Qilu Hospital, Shandong University, Jinan, China; 2 School of Stomotology, Shandong University, Jinan, China; 3 Key Laboratory of High Efficiency and Clean Mechanical Manufacture Ministry of Education, School of Mechanical Engineering, Shandong University, Jinan, China; 4 State Key Laboratory of Crystal Materials, Center of Bio & Micro/nano Functional Materials, Shandong University, Jinan, PR China; VIT University, INDIA

## Abstract

Endosseous titanium (Ti) implant failure due to poor biocompatibility of implant surface remains a major problem for osseointegration. Improving the topography of Ti surface may enhance osseointegration, however, the mechanism remains unknown. To investigate the effect of modified Ti surface on osteogenesis, we loaded rapamycin (RA) onto nano-hydroxyapatite (HAp) coated Ti surface which was acid-etched, alkali-heated and HAp coated sequentially. Sodium hyaluronate (SH) was employed as an intermediate layer for the load of RA, and a steady release rate of RA was maintained. Cell vitality of MC3T3-E1 was assessed by MTT. Osteogenesis of MC3T3-E1 on this modified Ti surface was evaluated by alkaline phosphatase (ALP) activity, mineralization and related osteogenesis genes *osteocalcin* (*OCN*), *osteopontin* (*OPN*), *Collagen-I* and *Runx2*. The result revealed that RA/SH-loaded nano-HAp Ti surface was innocent for cell vitality and even more beneficial for cell osteogenesis in vitro. Furthermore, osteogenesis of MC3T3-E1 showed significant association with the mammalian target of rapamycin (mTOR) phosphorylation by RA, which required further study about the mechanism. The approach to this modified Ti surface presented in this paper has high research value for the development of Ti-based implant.

## Introduction

Pure Ti and its alloys have been widely applied as endosseous implant in clinic due to their excellent biological and mechanical properties [1]. However, loosing implant and severe alveolar bone absorption are not rare in clinic. To improve the osseointegration on Ti-based implant, thus to prolong its service life, is one of the key issues to be resolved. During bone formation, osteogenesis related cells grow on the surface of Ti-based implant, and their biological behaviors are highly dominated by surface topography on meso-, micro-, and nano-scale sizes [2]. Accordingly, although some promising results have been reported, optimizing the surface composition and property for the improvement of the bioactivity of Ti-based implant is still needed [3].

To improve the bioactivity of Ti-based implant, various surface modification methods have been studied, such as plasma spraying [4], electrochemical treatment [5], chemical modification [6] and composite coating [7]. Among these methods, the art of coating on Ti-based implant is recognized to have the highest potential for mimicking micro- or nano-structure of human nature bone. The current state of this art includes biocompatible ceramics, bioactive proteins, ions and polymers [8–9]. HAp is one of the biocompatible ceramics that can be loaded on Ti-based implant [10]. Furthermore, various approaches to induce the formation of HAp layer improve the formation of osseointegration by regulating the adhesion and proliferation of osteoblast [11–12]. It is proposed that bonelike HAp can be formed on Ti-based implant surface in a simulated body fluid (SBF) with ion concentrations nearly equal to those of human blood plasma [13]. However, the synthesized layer usually binds unstably with Ti-based implant and does not enhance the osseointegration. Proper modification of Ti-based implant surface topography on micro-/nano- scale may improve this process. This means that the coated HAp on Ti-based material surface in SBF can mimic natural bone living environment, and experiments in vitro can be performed on it. However, the precise molecular mechanism of osteoblasts in response to this surface is still unclear. Therefore, identification of the critical signaling pathway involved in this phenomenon will lead to a further understanding of the mechanism how HAp coated Ti-based material surface affects osteogenesis.

mTOR pathway, which can be activated by growth factors and other extra cellular signals, regulates many major cellular processes including cell growth, proliferation, and survival [14]. Recent studies reveal that RA inhibited mTOR signaling improves osteogenesis on human stem cells [15]. According to the following studies, RA not only inhibits the proliferation of osteoblasts, but also induces the differentiation of osteoblasts and apoptosis of osteoclasts [16–17]. All these studies suggest that RA is a potential promoter for osteogenesis, but stable and suitable concentration in environment is crucial during this process. The nano-tubes system of HAp is promising for applications in various biomedical fields such as drug delivery [18–19], including delivering RA. However, due to the restriction of open HAp nano-tubes, a burst release of loaded RA is always happened on early phase in our previous study. For the stable loading and releasing of RA on HAp coated Ti-based materials, a third-party intermediary is needed. SH has been used extensively as an extracellular matrix coating molecule due to its cell proliferation activities [20]. The combination of SH and fp-151-RGD coated Ti foils improves MC3T3-E1 proliferation [21]. This paper investigates the combination of RA and SH (RA/SH) as a potential coating strategy on Ti-based materials for promoting osteogenesis.

The purpose of this study is to fabricate RA/SH-loaded nano-tubes of HAp on Ti-based materials and to investigate the synergistic effect of micro-/nano-topography and chemical cue (RA/SH) on the regulation of proliferation and differentiation of MC3T3-E1. We hypothesized that RA influenced MC3T3-E1 behavior in a sustained release SH/nano-HAp Ti system enhanced cells osteogenesis.

## Materials and methods

### Sample preparation

Ti foil (10x10x1mm^3^) was prepared from TC4 alloy (Al 5.5–6.75%, V 3.4–4.5%, Ti 88.1–91.1%). All foils were abraded and polished with SiC sandpaper ranging from 600 to 1200 grits to form polished Ti (PT) foil.

After being cleaned by ultrasonication in ethanol and distilled water for 15min, PT foil was successively etched in solution A (2mL 40%HF, 4mL 66%HNO_3_, add distilled water to 1000mL) at room temperature (RT) for 10min, in solution B (125mL 98%H_2_SO_4_, 121mL 37.5%HCl, add distilled water to 500mL) at 80°C for 20min and in solution C (50mL 98%H_2_SO_4_ and 50mL 30%H_2_O_2_) at RT for 60min. Then the etched foil was immediately rinsed with distilled water after every etching to form acid-etched Ti (ET) foil.

Alkali-heating treatment on ET and PT foil was performed by 20mL 10M NaOH in a high-pressure kettle at 100°C for 24h. After reaction was finished, the kettle was naturally cooled to RT, and then the foil was ultrasonicated in distilled water and dried to form alkali-heated PT (HT) and alkali-heated ET (NT) foil.

The formation of bonelike HAp layer was induced on the micro-structured surface of PT, ET, HT and NT (HAp/NT) foils in SBF, which had ion concentrations similar to human blood plasma as Kokubo et al did [13].

The surface topographies on Ti foils were inspected by scanning electron microscopy (SEM, Hitachi S-4800).The elements on Ti foils were identified and analyzed by energy dispersive X-ray spectrum (EDX, HORIBA, EMAX Energy EX-350).

### Drug load and release assay

1mg/mL SH was purchased from Furuida, China. RA and dimethyl sulfoxide (DMSO) were purchased from Sigma-Aldrich, USA. Ti foil was ultrasonicated in mixture solution (SH and RA, add DMSO to 10mL) for 10s and incubated at RT for 12h. Then, the foil was rinsed with phosphate buffer saline (PBS) to remove the physically adsorbed RA/SH and dried at RT. This process was repeated three times for following cell experiments.

The foil loaded with RA/SH was immersed into 1mL medium for MC3T3-E1 culturing in 24-well plates. 100μL degraded solution was taken from well for RA absorbance test at 320nm by spectrophotometric microplate reader (SMR, Biotek, USA) and 100μL fresh medium was added to maintain invariable culture volume within desired test time. The accumulated releasing amounts and percentages were calculated to evaluate the loading and releasing rate of RA on different Ti foils.

### Cell culture

Mouse osteoblastic MC3T3-E1 subclone 14 cell line was purchased from the Type Culture Collection of the Chinese Academy of Sciences, Shanghai, China (ATCC CRL-2594). MC3T3-E1 was cultured in a-MEM (Hyclone, USA) supplemented with 10% fetal bovine serum (FBS, Gibco, USA), 1% penicillin/streptomycin (Invitrogen, USA), and 2mM L-glutamine (Gibco, USA) and incubated in 5% CO_2_ atmosphere at 37°C. 200μL MC3T3-E1 suspension at a density of 2x10^4^ cells/well was seeded and rested for 2h on Ti foils in 24-well plate forcell attached on Ti foil not dish. Then 800μLfresh medium was added gently along the wall of each wells. MC3T3-E1 on Ti foil was cultured in medium for desired time points in all of the following experiments.

### Cell vitality assay

3-(4,5-dimethyl-2-thiazolyl)-2,5-diphenyl-2-H-tetrazolium bromide (MTT) was purchased from Genechem, China. The cell vitality was measured by MTT assay. At the prescribed time points, the Ti foils with cells were rinsed gently with PBS and transferred to a new 24-well plate. Then 100μL MTT solution (5mg/mL) was added and the specimens were incubated at 37°C for 4h. Afterwards, the formazen was dissolved using 500μLDMSO and the optical density (OD) was measured at 490nm on SMR.

### Total intracellular protein content assay

MC3T3-E1 was lysed by distilled water with three freeze-thaw cycles. Total intracellular protein content in the cell lysates was measured at 570nm on SMR and assessed from a standard curve of absorbance which was measured with BCA kit (Beyotime, China).

### Alkaline phosphatase (ALP) activity assay

MC3T3-E1 was harvested in 1mL distilled water, ultrasonicated for 10min and centrifuged at 2000 rpm in ice bath for 10min. The ALP activity (expressed as μmol of converted p-nitro-phenol/min) was measured at 405nm on SMR and normalized by the total intracellular protein production.

### Mineralization ability

Formaldehyde, alizarin red-S (ARS), acetic acid, mineral oil, ammonium hydroxide were all purchased from Sigma-Aldrich, USA.Ti foils with cells were washed with PBS and fixed in 10% formaldehyde at RT for 15min. After being washed with distilled water, Ti foils were immersed into 1mL 40mM ARS with pH 4.1 per well for staining at RT for 20min. Next, 400μL 10% acetic acid was added to each well and cells were transferred to a 1.5mL tube. Sonicating cells in ice bath for 2min and removing the supernatant to a new tube. Mixing 500μL mineral oil to supernatant and heating at 85°C for 10min, and cooled in ice bath. Centrifuging mixture solution at 2000 rpm for 15min and removing supernatant to a new tube. Then 10% ammonium hydroxide with equal volume of supernatant was added to neutralize the acid. The mineralization ability was quantified by absorbance at 405nm on SMR.

### RNA isolation and gene expression by RT-PCR analysis

Total RNA was isolated from cells cultured after desired days by Trizol Reagent (Gibco, USA). cDNA fragments were amplified using SYBR Green chemistry (Roche Applied Science, Germany) in an ABI PRISM 7900 instrument (AppliedBiosystems, USA) according to previous study [22].Highly purified gene-specific primers for *OCN*, *OPN*, *Collagen-I*, *Runx2* and *GAPDH* were synthesized commercially (Genechem, China). Primers sequences used were as follows: *GAPDH*: 5'-TTGTCTCCTGCGACTTCAACA-3'; 5'-GTGGTCAGGGTTTCTTACTCC-3';*OCN*:5'-TGGCCCTGAC TGCATTCTGC-3'; 5'-GCTGTGCCGTCCATACTTTCG-3';*OPN*:5'-CCTCTGAAGAAACGGATGACT-3'; 5'-CTGTGTGTTTCCACGCTT-3'; *Collagen-I*:5'-CGAGTATGGAAGCGAAGGTT-3'; 5'-CACAAGCGTGCTGTAGGT-3'; *Runx2*:5'-CGCATTCCTCATCCCAGTAT-3'; 5'-TGTCCACGAAGTCTTGACCC-3'. Each mRNA value was normalized to that of *GAPDH*. Results are reported as relative gene expression.

### Western blotting analysis

All of the antibodies were purchased from Abcam, UK. Protein assay kit and ECL reagent were purchased from Beyotime, China. After culturing for desired days, the total proteins in cells were extracted by lysis buffer, and the lysates were resolved by SDS-PAGE and transferred to PDVF membranes using protein assay kit.The membranes were incubated with the primary antibodies (Abcam, UK) diluted in TBST (20mM Tris, 135mM NaCl, and 0.05% Tween 20)and then with HRP-conjugated secondary antibodies followed by exposure to ECL reagent. The quantitative analysis of immunoblots bands was performed with Quantity One (Bio-Rad, USA).

### Statistical analysis

All data were expressed as mean standard deviation from at least three independent experiments. They were analyzed by student’s t-test and one-way analysis of variance (ANOVA) using SPSS 19.0 software. Multiple comparison between the groups was performed using S-N-K method. *P*<0.05 was considered statistically different. *compared with sample on PT. # compared with sample on NT. Δcompared with sample on HAp/NT.

## Results

### Surface characterization

**Fig 1:**
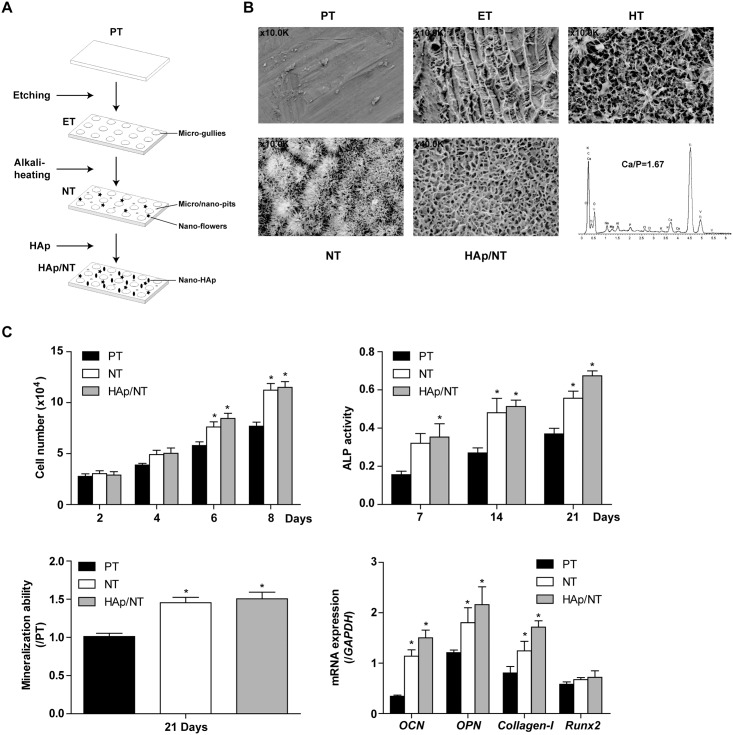
The modification of topography on Ti foils changes the osteogenesis of MC3T3-E1. 1A shows the fabrication scheme of nano-scale HAp onto the surface of modified Ti foils; B shows the surface topography by SEM on each approach and by EDX on HAp/NT foils; C shows cell vitality and osteogenesis of MC3T3-E1 on PT, NT and HAp/NT surface, which are evaluated by ALP activity, mineralization and related osteogenesis genes *OCN*, *OPN*, *Collagen-I* and *Runx2*.

The surface morphologies of modified Ti foils were processed (Fig 1A) and characterized by SEM (Fig 1B). The PT foils possessed no apparent micro/nano-topography. The ET foils showed no obvious nano-topography, but possessed micro-gullies as indicated in 10.0K magnification image. The HT foils showed micro-pits compared with micro-gullies on ET foils. The NT foils were composed of micro/nano-pits and nano-flowers, which were considered to be appropriate substrate for nano-HAp coating. After several trials, we could not find any HAp topography on PT, ET or HT via this method. With the coating of HAp on NT foils, they had apparent nano-HAp pits topography in 40.0K magnification image. EDX of HAp/NT foils showed the ratio ofCa to P was 1.67, which indicated that HAp (Ca_10_(PO_4_)_6_(OH)_2_) was coated on TiO_2_ surface.

### Topography influences MC3T3-E1 osteogenesis on Ti foils

Compared to PT, both of NT and HAp/NT gave rise to cell vitality on the 6th and 8th day due to the micro/nano-topography (Fig 1C). The differentiation of MC3T3-E1 on Ti foils was assessed in terms of ALP activity, mineralization ability and osteogenesis related gene expression. The ALP activities of all three groups were started to ascend on the 7th day and reach the peak on the 21st day. Cells grown on NT and HAp/NT surfaces showed statistically higher ALP activities than those on PT surface at each time points. The cell mineralization result showed a similar trend among different surfaces. On the 21st day, NT and HAp/NT mineralized much more than PT. The expression of osteogenesis related genes including *OCN*, *OPN*, *Collagen-I* and *Runx2* after 2 days culture were monitored by RT-PCR. PT induced slightly lower expression than NT and HAp/NT for *Runx2*. For *OCN*, *OPN* and *Collagen-I*, PT induced significantly lower expression than NT and HAp/NT. There were insignificant differences between NT and HAp/NT surfaces on MC3T3-E1 cell vitality, ALP activity, mineralization and expression of osteogenesis related genes.

### Optimization of RA/SH compositions for drug load/release system and MC3T3-E1 osteogenesis on Ti foils

**Fig 2:**
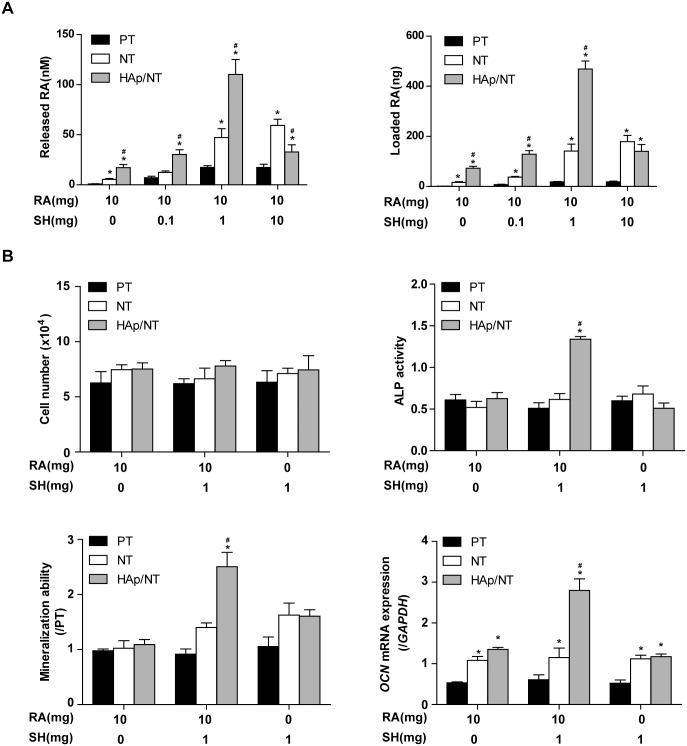
Various RA/SH compositions on Ti foils changes the osteogenesis of MC3T3-E1. 2A shows the released RA concentration at 72h and loaded RA amounts on various RA/SH compositions on PT, NT and HAp/NT; B shows cell vitality and osteogenesis of MC3T3-E1 on various RA/SH compositions on PT, NT and HAp/NT, which are evaluated by ALP activity, mineralization and related osteogenesis genes *OCN*.

As an important factor in drug release system, SH acts a connector between RA and nano-HAp tubes. To optimize the proportion of RA/SH, gradient SH (0, 0.1, 1, 10mg) mixed with constant RA (10mg) in 10mL DMSO were performed on PT, NT and HAp/NT foils. These foils were immersed into medium for 30 days, during which medium was changed once every three days and quantified the current concentration by SMR to calculate the accumulated loaded RA. The result showed that RA could not load on any Ti foils regardless of the percentage of SH. At the composition of 10mg RA and 1mg SH in 10mL DMSO, both of released concentration and loaded amount on HAp/NT foil were significantly higher than those on other foils (Fig 2A). Accordingly, we demonstrated that 1mg SH improved the loading and releasing ability of 10mg RA. To further evaluate the relationship of 10mg RA and 1mg SH in 10mL DMSO on cell vitality and differentiation, respectively, MTT, ALP activity, mineralization ability and PCR for *OCN* mRNA expression were performed. The result demonstrated that the composition of 10mg RA and 1mg SH increased remarkably the ALP activity on the 7th day, mineralization ability on the 21st day and *OCN* mRNA expression on the 2nd day (Fig 2B). However, none compositions had any effect on cell vitality.

### RA/SH influences drug load/release system on HAp/NT foils

**Fig 3:**
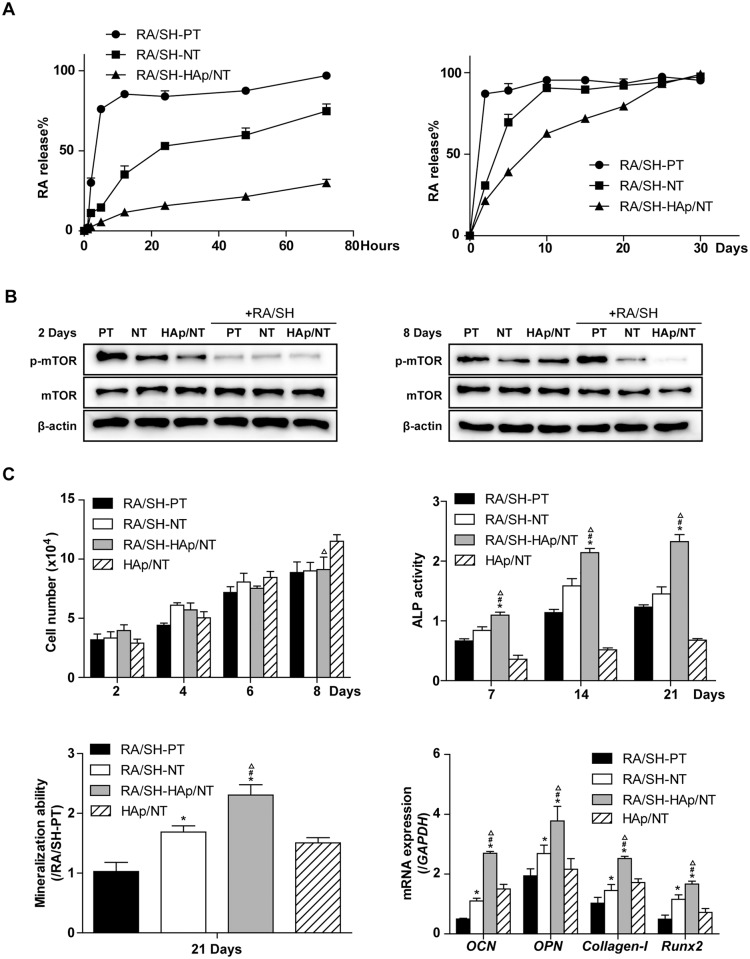
The modification of topography on Ti surface changes the osteogenesis of MC3T3-E1 via mTOR signaling pathway. 3A shows the RA release rate of RA/SH binding PT, NT and HAp/NT surface during short- and long-term culture; B shows the expression of p-mTOR and mTOR with or whithout RA/SH binding PT, NT and HAp/NT surface on the 2nd and 8th day; C shows cell vitality and osteogenesis of MC3T3-E1 on RA/SH binding PT, NT and HAp/NT surface, which are evaluated by ALP activity, mineralization and related osteogenesis genes *OCN*, *OPN*, *Collagen-I* and *Runx2*.

Another important issue for these HAp/NT foils was the delivery of the target drug during short- and long-term cell culture by comparing with PT and NT. We hypothesized that the HAp/NT could provide many biochemical cues to generate an artificial niche for drug release during short- and long-term cell culture. To test the hypothesis, RA/SH (1mg:10mg) was loaded in nano-HAp tubes and cultured with MC3T3-E1 up to 30 days, and its release was compared to PT and NT under the same conditions. The results of cumulative release of RA from PT, NT and HAp/NT were compared in Fig 3A. RA was dramatically released from controlled PT and NT with 96.9% and 74.7% respectively in total after cultured for 72h, respectively. In initial 12h, the PT showed a burst release of 85.4% RA. After 72h incubation for PT and 10 days for NT, the releasing rate of RA reached a plateau, when few RA remained in PT or NT. It seemed that RA would not totally be released unless being ultrasonically cleaned. Comparing to these, the HAp/NT foils exhibited significantly continuous and stable release behavior as expected, which showed that 79.3% of RA loaded in nano-HAp tubes was released after 20 days (*P*<0.05) and further releasing rate was thus significantly decreased. One could speculate that the nano-tubes on HAp/NT sustained more effective RA release than that of micro/nano-structure on NT and PT.

### RA/SH influences p-mTOR and mTOR expression of MC3T3-E1 on HAp/NT foils

We evaluated the sensitivity of cells to RA/SH by monitoring the levels of mTOR phosphorylation on Ti foils. The cells were cultured on PT, NT and HAp/NT which loaded RA/SH or not. Whole cell lysates prepared from untreated cells and RA/SH-treated cells were analyzed by western blotting using antibodies against mTOR, phosphorylated mTOR (p-mTOR) and β-actin (as control) (Fig 3B). The result showed that the levels of p-mTOR and mTOR were not changed by topographies on different Ti foils surfaces, suggesting that micro-/nano-topography had no significant effects on mTOR dependent pathway in cells. With treatment of loaded RA/SH for 72h, the mTOR pathway was potently suppressed in cells, as demonstrated by decreased expression of p-mTOR on NT and HAp/NT. The expression of p-mTOR on HAp/NT decreased more than that on PT and NT, due to the sustained release of RA. The total protein levels were not significantly changed with RA/SH treatment.

### RA/SH influences MC3T3-E1 osteogenesis on HAp/NT foils

The time point of RA released out was the 3rd day on PT, the 10th day on NT and the 25th day on HAp/NT (Fig 3A). The cell vitality result assessed by MTT showed insignificant differences among PT, NT and HAp/NT surfaces, which indicated that RA/SH loaded on foils reduced the cell vitality after six-day culturing. The ALP activity and mineralization abilityshowed obviously increase on RA/SH loaded HAp/NT compared to that on PT and NT. To assess the long-term effect of RA/SH on osteogensis related genes, MC3T3-E1 was cultured on Ti foils for 14 days. The result showed that continuous and stable release of RA/SH increased the expression of osteogensis related genes on RA/SH-HAp/NT compared to that on other Ti foils. It was demonstrated that RA/SH enhanced ability of osteogenesis but reduced cell vitality of MC3T3-E1 on Ti foils.

## Discussion

The treatment of Ti by HCl and subsequent NaOH is a suitable method for apatite formation on this chemical treated Ti surface [23]. Firstly, acid etching of Ti is an alternative pretreatment to obtain a uniform initial micro-topography Ti surface before alkali treatment. More importantly, there is a high bonding strength of HAp coating to alkali-heat treated Ti substrate [24]. Then, the formed NT surface improves the corrosion-resistance properties of Ti substrates and contributes to the growth of HAp on this surface [25]. In this study, we further perform PT by etching on acid mixture and subsequently by alkali-heat treatment, which forms micro/nano-topography such as micro/nano-pits and nano-flowers. The morphology of nano-flower is several separate dendrites growing outward in certain directions from the main trunk. The dendrites become sharper while growing from the trunk to form tips outside, just like petals of a flower, which is a good substrate for mineral deposition [26]. This result supports that apatite is successfully deposited onto micro/nano-topography of NT surface and then bone-like HAp nano-topography is constructed.

There are many drawbacks of general drug therapy in bone, such as over administration, long course, inefficiency, poor bioavailability and toxicity. Ideally, in order to improve efficiency and to minimize side effects, drugs should be applied contrapuntally and specifically to bone sites requiring therapy at optimal concentrations and over appropriate time periods [27–28]. Toward precision medicine for drug-delivery to an individual, local drug delivery should be recognized as a promising solution. HAp drug-delivery systems have been limited due to the difficulty in loading drugs that are insoluble in water [29]. Therefore, HAp nano-particles are considered to be beneficial as a nano-carrier. Nano-carriers are desirable for both systemic and localized delivery due to the increased circulation time and the capacity to transport hydrophobic materials [30].

SH is an almost essential component of extracellular matrices. Early in embryogenesis mesenchymal cells migrate, proliferate and differentiate, in part, are influenced by SH. Since the features of embryogenesis are revisited during wound repair, including bone fracture repair, SH is recognized as a potential promoter for osteogenesis [31]. In previous study, our group proves that the drugs are wrapped in SH or adsorbed on its surface as drug carrier into cell by endocytosis [32]. RA is one of hydrophobic drugs that were difficult to maintain constant concentration in cell culture medium or to be loaded a large amount on materials. In this study we evaluate whether SH has an effect on loading or releasing RA for osteogenesis on Ti foils. This experiment finds that the cross-linking of SH and RA optimizes the loading and releasing rate of RA on HAp/NT, due to SH enhances hydrophilicity and moisture of RA. Furthermore, SH conducts a viscous solid-liquid conversion, which further enhances the mechanical strength of Ti foils and stability of RA.

In previous study, our group modified topography of Ti surface to achieve bioactive Ti for dental implant, bone screws and plates [33]. Our result proves that both micro- and nano-topographies improve cell functions on osteogenesis. The possible reason is that the dramatically increased surface-area-to-volume ratio promotes osteogenic differentiation and cell proliferation. To investigate the mechanism of this phenomenon, we tested the expression of mTOR and activated mTOR (p-mTOR) of MC3T3-E1 on PT, NT, HAp/NT with or without RA/SH loaded on the 2th and 8th day. The result proves that the increased osteogenic differentiation and cell proliferation are closely related to the phosphorylation of mTOR, and further concludes that mTOR signaling pathway may participate in the osteogenesis of MC3T3-E1 on Ti surface. Although the proliferation of MC3T3-E1 is slightly inhibited by RA, the enhanced cell vitality by nano-HAp topography is balanced against it. More impartant, there is continuous increase on osteogenesis on RA/SH loaded nano-HAp Ti foils. The possible mechanism of RA on Ti improves osteogenesis requires further study.

## Supporting information

S1 Table Original data of Fig 1. (XLSX)

S2 Table Original data of Fig 2. (XLSX)

S3 Table Original data of Fig 3. (XLSX)
